# Developments in breeding of *Agaricus bisporus* var. *bisporus*: progress made and technical and legal hurdles to take

**DOI:** 10.1007/s00253-017-8102-2

**Published:** 2017-01-28

**Authors:** Anton S.M. Sonnenberg, Johan J. P. Baars, Wei Gao, Richard G.F. Visser

**Affiliations:** 1 Plant Breeding, Wageningen University and Research, 6708 PB, Wageningen, Netherlands; 2 Institute of Agricultural Resources and Regional Planning of CAAS, Zhongguancun South Street 12, Beijing, 100081 China

**Keywords:** Button mushroom, *Agaricus bisporus*, Breeding, Essentially derived varieties, Strain protection

## Abstract

True breeding of button mushrooms has hardly been done in the last decades, despite this species being one of the most cultivated mushrooms worldwide. Research done in the last 20 years has identified and characterised new germplasm and improved our understanding of the genetic base for some traits. A substantial collection of wild-collected strains is now available and partly characterised for a number of important traits such as disease resistance and yield. Most of the variations found in a number of important agronomic traits have a considerable heritability and are thus useful for breeding. Genetic marker technology has also developed considerably for this mushrooms in the last decade and used to identify quantitative trait loci (QTL) for important agronomic traits. This progress has, except for one example, not resulted so far into new commercially varieties. One of the reasons lies in the typical life cycle of the button mushroom *Agaricus bisporus* var. *bisporus* which hampers breeding. Joint investment is needed to solve technical problems in breeding. Special attention is needed for the protection of new varieties. Due to its typical life cycle, it is very easy to generate so called “look-a-likes” from protected cultivars by screening fertile single spore cultures. A consensus has been reached within the mushroom (breeding) industry to consider this method as the generation of essentially derived varieties as defined in plant breeding.

**Electronic supplementary material:**

The online version of this article (doi:10.1007/s00253-017-8102-2) contains supplementary material, which is available to authorized users.

## Introduction

The button mushroom (*Agaricus bisporus* var. *bisporus*) is one of the most cultivated mushrooms in the world. It is the predominant mushroom cultivated in Western countries and the cultivation area is expanding fast in China (Zhang et al. 2014). Progress has been made in recent years in various areas essential for breeding, such as collection and characterisation of source material, development of molecular markers, linkage mapping and detection of quantitative trait loci (QTL). However, these efforts have hardly led to new cultivars during the last three decades. The main reason lies in the typical life cycle of this fungus which hampers the introduction of traits without considerable linkage drag. Its typical life cycle also facilitates the easy generation of essentially derived varieties. The latter can outcompete a cultivar generated by cross breeding and thus minimise the investment done by that breeder. In recent years, only one genuinely new button mushroom cultivar has been marketed successfully in Western countries, a brown cultivar generated by Amycel, an American breeding/spawn producing company. This variety has been generated by cross or outbreeding using at least three different strains. Due to the previously mentioned difficulties to protect new varieties, Amycel has chosen to protect this variety in a more strict way, i.e. utility patent in the USA, and it is in process in trying to obtain a similar patent in other countries around the world.

In this paper, we will review the state of the art of different aspects of breeding and breeding research for button mushrooms and will specifically address the main technical and legal hurdles that exist for breeding button mushrooms and discuss actions that are taken or need to be taken to solve these.

## The typical life cycle of *A. bisporus* var. *bisporus*

All commercial and most wild-collected strains have a secondary homothallic life cycle (Raper et al. 1972; Xu et al. 1998). This variety has a unifactorial mating system and the A-mating locus is mapped on the largest linkage group, chromosome I (Xu et al. 1993). Meiosis takes place in specialized cells (basidia) on lamellae where the two haploid nuclei fuse and meiosis I and II lead to the formation of four recombinant haploid nuclei (Kerrigan et al. 1993). The majority of the basidia produce only two spores and non-sister nuclei are preferably paired into one spore (Summerbell et al. 1989). This intra-tetrade mating leads to the formation of spores that germinate into heterokaryons containing nuclei with different mating types, a prerequisite to produce fruiting bodies. Only a minority of the basidia produce three or four spores which will generate homokaryons containing one type of haploid nucleus. These homokaryons need to be mated with compatible homokaryons (with a different mating type) in order to produce mushrooms. Such homokaryons are thus useful for outbreeding. Homokaryotic single spore isolates (SSI) show in general a lower growth rate than heterokaryotic SSI (Kerrigan et al. 1992) and this character is often use to preselect for homokaryons in spore prints. Confirmation of the homokaryotic status is subsequently confirmed with genetic markers (Gao et al. 2013). Attempts to enrich for homokaryons from spore prints using cytometric tools such as cell sorting lead to some enrichment but not better than the preselection on growth rate. Next to the additional effort needed for the selection of homokaryotic SSI, breeding of bisporic varieties is also hampered by its typical recombination landscape. Crossovers (CO) are mainly restricted to chromosome ends and >90% of these CO are found within a 100-kb distance of the chromosome ends (Sonnenberg et al. 2016). The largest part of each chromosome remains thus parental type after meiosis. The predominant pairing of non-sister nuclei in most basidia in combination with CO restricted to chromosome ends leads to heterokaryotic spores that have a genetic make-up that is very similar to its parent. As a result, they have also phenotypes very similar to its parent with only one or a very few changes in traits.

## The origin of button mushroom cultivars

**Fig. 1:**
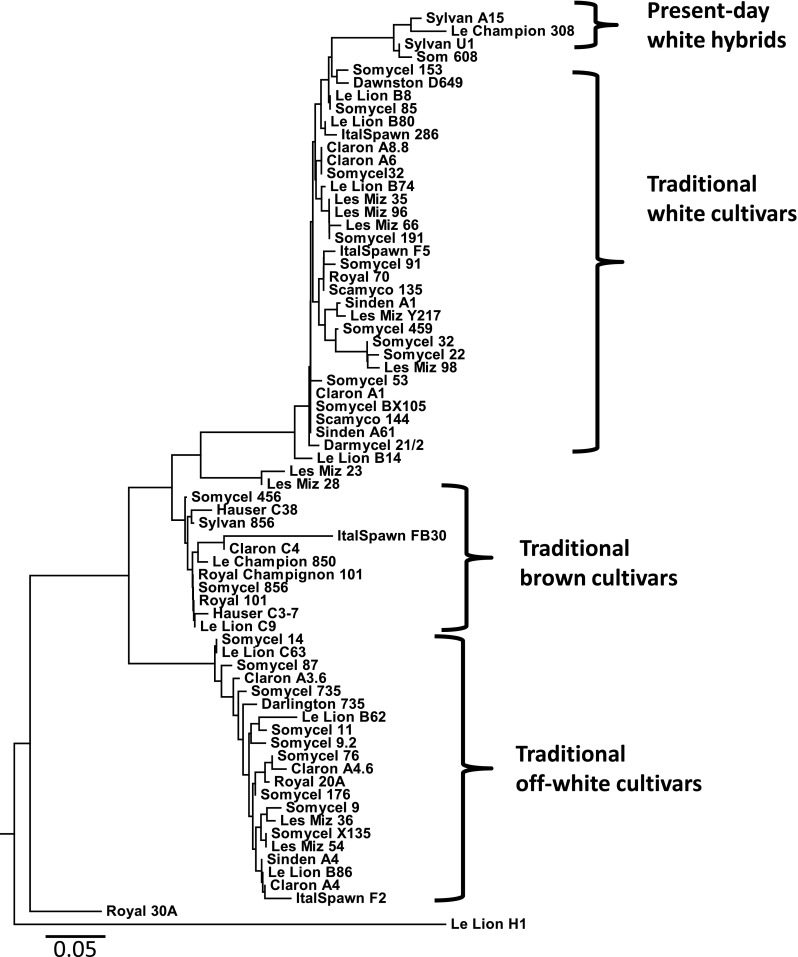
Dendrogram of traditional and present-day button mushroom cultivars. Strains were genotyped using 115 SNP markers (supplementary file S1). A clear clustering is seen for each type of strain indicating that all strains within each cluster are copies or essentially derived varieties from each other

**Fig. 2:**
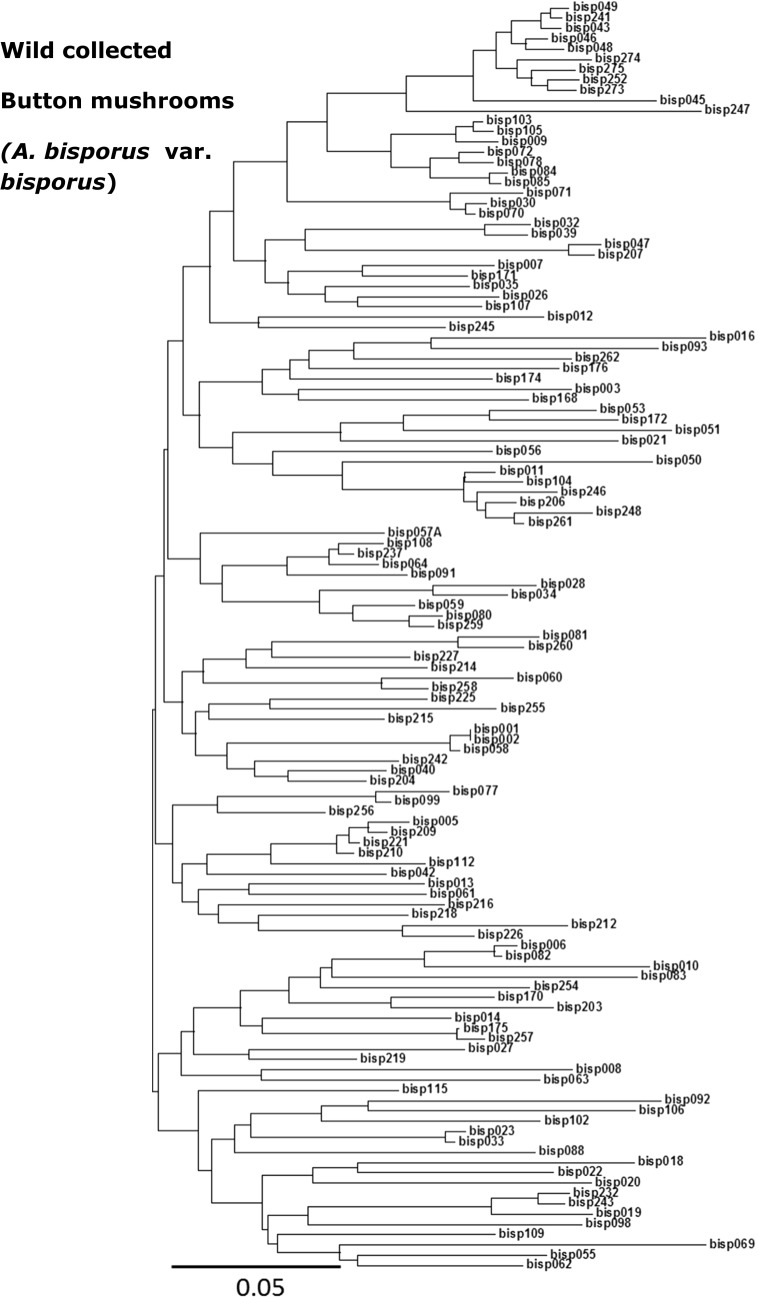
Dendrogram of wild button mushroom *Agaricus bisporus* var. *bisporus*. Strains were genotyped using 115 SNP markers (supplementary file S1). Wild-collected strains show a clearly higher genetic diversity compared to cultivars (Fig. 1)

Breeding by outcrossing is relatively new in the button mushroom industry. Before the first strains produced by outcrossing reached the market, new cultivars were normally generated from existing cultivars by selecting heterokaryotic SSI or by generating multi-spore cultures (Elliott and Langton 1981; Fritsche 1981). Mainly three types of cultivars were used at that time for mushroom production, white, off-white and brown strains (Fritsche 1981). Only in 1980, the first white hybrids were introduced into the market that were generated by crossing homokaryons derived from different cultivars (Fritsche 1982). One of these varieties, Horst U1, became in short time the main commercial white cultivar in many countries. It has been generated by a cross between a homokaryon of the white cultivar Somycel 53 and a homokaryon from the off-white cultivar Somycel 9.2 (Fritsche 1982). Within the relatively short period of a few years, many other white “hybrid” varieties appeared on the market. These were genetically and phenotypically identical or very similar to this first hybrid. A thorough genotyping of many of these varieties showed that they were all derived from the first hybrids by selecting fertile single spore cultures (Sonnenberg et al. 2016). We have genotyped the traditional cultivars, some of the present-day hybrids and the wild bisporic strains, using SNP markers derived from several resequenced lines. The genotyping methodology, sequence of SNP markers and strains used are described in the supplementary data (Tables S1-4). The analysis of these data shows that the traditional and present-day cultivars cluster into the expected groups with a very low genetic variation within each group (Fig. 1). This contrasts with the wild isolates that show a broad genetic variation (Fig. 2) and clearly shows that the selection of fertile single spore cultures of the *bisporus* varieties has been a common breeding practice for a long time and most probably still is.

A few decades ago, a distinctive variety was described, firstly found in the Sonoran Desert in California (Callac et al. 1993) and later also in France (Callac et al. 1998a). It has predominantly four-spored basidia (>90% of basidia bear four spores). This variety was designated as *A. bisporus var. burnetti*, named after the first collector of this variety. After the description of this new variety, the common button mushrooms with the predominant secondarily homothallic life cycle have been designated as *A. bisporus* var. *bisporus*. These two varieties are fully compatible. Segregation analyses of offspring from *bisporus* x *burnettii* hybrids indicate that the recombination landscape in the *burnettii* variety is normal, i.e. more evenly spread over the whole chromosome (Callac et al. 1997; Foulongne-Oriol et al. 2010).

## Global genetic resources for *A. bisporus*

Isolates from nature in many geographic origins have now been collected, described and preserved (Kerrigan et al. 1995). Especially from North America, Europe and western Asia, indigenous non-cultivar populations have been found that are available through the Agaricus Resource Program, an initiative by Rick Kerrigan, working at Sylvan Inc., Kittanning, USA (Kerrigan 1996). There are also well-documented collections of apparently native *A. bisporus* from other parts of the world, including Central Mexico, Morocco, Zaire, New Zealand, China, Australia and Tasmania (Kerrigan 2004). The South American countries and Southern Africa are areas that seem not to have been sampled at all. Additional wild isolates have been collected, described and preserved from France and Greece (Callac 1995, 2002), but these strains are not publically available. A number of reports describe the frequent presence of cultivar types in nature in certain areas (sometimes up to 50% of the sampled strains) and the threat this might pose on the indigenous populations (Xu et al. 1997; Kerrigan et al. 1998). Also, the change in natural biotopes for *A. bisporus*, such as the disappearance of cypress trees, forms an additional threat. There is thus a substantial collection of resources available for breeding purposes but very likely representing only a small part from the diversity that is present in nature at large. Except for scarce reports on wild isolates (Mata et al. 2016; Sobieralski et al. 2010; Rokni et al. 2015), there are no efforts known at this moment to extend the collections, which is mainly due to lack of funding.

## Characterisation of collections for target traits in breeding

Whereas the present-day commercial cultivars are almost identical in all traits, a large variation in characters has been found in wild-collected strains. Resistance or high tolerance against the common diseases such as the bacterial blotch (Moquet et al. 1999) and the fungal pathogen *Lecanicillium fungicola* (Dragt 1995; Largeteau 2004; Sonnenberg 2005) was found in wild strains, especially in strains of the var. *burnettii*. The heritability of resistance to both diseases are substantial (Moquet et al. 1999; Foulongne-Oriol et al. 2011b) indicating potential for breeding. A fungal disease caused by *Trichoderma aggressivum* f. *europeaeum* was first reported in the UK and Ireland around 1988 (Seaby 1996), and a few years later, a similar strain—*T. aggressivum* f. *aggressivum*—emerged independently in Canada and the USA. In the years that followed, the disease spread into France and other European countries (Hatvani et al. 2007) as well as in Mexico and in the Americas. So far, no resistance has been found against this disease in wild-collected strains although tolerance to some degree might be present (Chen et al. 2003; Kerrigan 2004; Sobieralski et al. 2010; Foulongne-Oriol et al. 2011b). Two viral diseases have been described for the button mushroom, i.e. La France or Die Back disease (Hollings 1962) and Mushroom Virus X (Gaze et al. 2000) both consisting of a complex of double-stranded RNAs (ds-RNAs). There are indications that there is immunity for La France disease in some wild isolates (Sonnenberg et al. 1995) since transmission of La France to these strains by anastomosis or mating did not result in transmission of ds-RNAs, whereas this could be done repeatedly between commercial lines. Fu et al. (2016) tested 17 wild-collected strains of *A. bisporus* (mainly from the Sichuan and Fujian regions in China) and 11 commercial strains for their susceptibility to wet bubble disease (caused by *Mycogone perniciosa*). They found 10 out of 17 wild-collected strains to be highly resistant to infection by *M. perniciosa*. Among the commercial strains, they found one to be highly resistant, two to be moderately resistant and seven to be highly susceptible. As far as we know, no resistance or tolerance to other minor diseases has been reported for wild-collected strains. Next to disease resistance, some wild strains are also less sensitive to discoloration after damage of the cap skin (Gao et al. 2013). This trait is very useful in generating cultivars that can be harvested mechanically (low bruising sensitivity) and cultivars that have a better shelf life.Despite the differences between their habitats, most wild strains are able to produce mushrooms on conventional substrate (Savoie et al. 1996; Kerrigan 2004) although assessment of enzyme activities involved in substrate utilisation indicated that the cultivated strains are better adapted to the conventional substrate (Savoie et al. 1996). Next to the predominantly four-spored basidia and an apparently normal recombination landscape, most *burnettii* strains are able to produce mushrooms at an elevated temperature, i.e. 25 °C instead of 16–19 °C, optimal for all commercial cultivars (Largeteau et al. 2011). This offers opportunities to breed cultivars for warmer regions or cultivation at higher temperatures in summer periods, thus reducing energy costs.

## Genetic markers and genetic dissection of traits

As for other crops, the type of genetic markers has developed from isozyme up to the now common single nucleotide polymorphic markers (SNPs; for an overview on markers used in linkage analysis in fungi, including *A. bisporus* see: Foulongne-Oriol 2012). May and Royse (1981, 1982) started to use isoenzyme markers to elucidate the life cycle of *A. bisporus* and confirm crosses between homokaryons. Later, RAPD markers were used (Khush et al. 1992; Moore et al. 2001) mainly to identify button mushroom cultivars illustrating the narrow genetic base of the past and present button mushroom cultivars. Restriction fragment length polymorphism (RFLP) markers were used to both elucidate the life cycle of *A. bisporus* (Summerbell et al. 1989), to genotype commercial and wild lines (Loftus et al. 1988) and to generate the first linkage map (Kerrigan et al. 1993). Foulongne-Oriol et al. (2009) demonstrated that microsatellite markers were more powerful to generate linkage maps for *A. bisporus*. Since the publication of the whole genome sequence of the button mushroom (Morin et al. 2012; Sonnenberg et al. 2016), SNP markers can be used and they appear to be very useful in generating linkage maps (Gao et al. 2016, 2015) and for precise location of meiotic crossovers (Sonnenberg et al. 2016).

A number of studies have been done on the genetic base of useful phenotypic variations found in wild strains using a range of genetic markers. Crosses between var. *bisporus* and var. *burnettii* have been used to map the main determinant for the number of spores on basidia (predominantly two- or four-spored). The four-spored trait appeared to be dominant to some extent with the main genetic determinant located on chromosome I (Imbernon et al. 1996). The cap color was mapped to chromosome VIII (Callac et al. 1998a; Callac et al. 1998b) and interpreted as a single recessive allele for the white color. Later work showed that additional minor QTL for color are located on chromosomes II and VII (Foulongne-Oriol et al. 2012a; Gao et al. 2015). For most crops, agronomic traits are complex and controlled by a number of genes. The button mushroom is no exception to this. QTL were found for complex traits as yield, disease resistance and shelf life (discoloration). Foulongne et al. (Foulongne-Oriol et al. 2012a; Foulongne-Oriol et al. 2012b) found 23 QTL related to yield components in a segregating offspring between a *bisporus* x *burnettii* cross explaining ca. 21% of the variation in yield. This population was also used for quantitative genetics on resistance to the fungal pathogen *L. fungicola* (Foulongne-Oriol et al. 2011b). The study showed that the best description of the disease progress is done by a separate assessment of the two main symptoms (dry bubbles and spots), for which likely independent mechanisms are involved differently affected by the environment. Tolerance to *L. fungicola* was thus found to be a polygenic trait with low sensitivity mainly contributed by the var. *burnettii*. Moquet et al. (1999) mapped resistance to bacterial blotch and found a QTL linked to the cap color explaining 30% of the variation in resistance. Gao et al. (2015) found QTL for sensitivity for bruising (discoloration after damaging the cap skin). The trait appeared to be highly heritable and the major QTL explained ca. 50% of the variation. Gao et al. (2016) also analysed the quality traits with five additional agronomic traits through multi-trait QTL analyses in a mixed model. Major and minor QTL were detected for the trait earliness (first day picking), firmness, cap color, compost colonisation and scaling of mushroom caps. For a number of important agronomic traits, variations are thus present in wild-collected strains and these variations have a considerable heritability and are thus useful for breeding. The markers used to map these traits can thus in principle be used to introduce new traits in existing cultivars by marker-assisted selection.

## Obstacles in breeding

As mentioned in a previous paragraph, the button mushroom is mainly represented by two varieties, i.e. var. *bisporus* and var. *burnettii*. The former produces mainly two-spored basidia and recombination in meiosis is restricted to chromosome ends, whereas the latter produces mainly four-spored basidia and has a seemingly even distribution of recombination over the entire chromosome. For breeding purposes, the *burnettii* variety is obviously the preferred variety since isolation of homokaryotic single spore cultures (SSI), used for outcrossing, is less cumbersome and traits can be mapped more precisely and introduced into germplasm without substantial linkage drag. All commercial cultivars and most wild isolates represent the *bisporus* variety. Despite the beneficial traits mentioned in the previous paragraph, the average quality of most *burnetti* strains is poor. Especially the production of abundant numbers of small mushrooms that show a quick maturation (cap opening) is considered as poor quality (A.S.M. Sonnenberg, unpublished observations). The *bisporus* variety will thus remain for the time being the main platform to produce new varieties. The obvious preliminary breeding activity would be the introduction of the “breeding-efficiency” traits (tetrasporic, recombination evenly across the chromosome) of the *burnettii* variety into a *bisporus* breeding stock. That might require some investment since each of these traits seems to be controlled by more than one gene. Foulongne-Oriol et al. (2011a) compared genetic linkage maps using a haploid offspring of an intervarietal cross *bisporus* x *burnettii* (H population) with a map generated by an offspring of a cross between a homokaryon of the H population backcrossed with a homokaryon of a *bisporus* variety (designated as the Z population). The map generated with the Z population was shorter (851 cM) than the map generated with the H population (1156 cM). The authors explain this by a reduction of the recombination frequency due to the backcross with the *bisporus* homokaryon. This reduction in observed recombination can also be explained by the fact that a backcross with a *bisporus* homokaryon shifts recombination more towards chromosome ends where recombination is less efficiently detected (Sonnenberg et al. 2016). This suggests that the trait “recombination landscape” is quantitatively inherited and likely polygenic and thus complex. Also, the trait basidial spore number (BSN) appears to be complex although less than the trait recombination landscape. BSN exhibits dominancy with a variable degree of expression (Kerrigan et al. 1994). The main genetic determinant for BSN was mapped on chromosome I (Imbernon et al. 1996). Attempts to retain the elevated basidial spore numbers by selecting for markers linked to BSN in backcrosses to *bisporus* varieties resulted in a gradual decrease of the average number of spores per basidium (N. Sedaghat-Tellgerd, Plant Breeding, WUR, pers. com). That indicates that also for BSN, more than one gene is involved. The introduction of both of these traits in all *bisporus* breeding stock will fix these traits and certainly facilitate breeding.

## Mushroom breeding versus plant breeding

Compared to plant breeding, (button) mushroom breeding has advantages but also drawbacks. In plants, haploid nuclei fuse immediately after fertilisation, whereas in most edible fungi, the nuclei remain side by side after mating of homokaryons. Only just before spores are formed in mushrooms does nuclei fuse and undergoes meiosis. That allows the recovery of the constituent nuclei as homokaryons and thus genotyping of the haplotypes. The haploid SSI are similar to gametes in plants and contrary to plant gametes, these single spore cultures can be propagated and genotyped. Linkage maps can thus be generated directly on meiotic products. In some occasions, a genome of plant gametes can be doubled (double haploids) allowing the generation of plants and thus phenotyping to map quantitative traits directly on haplotypes. That is not possible in fungi. The haploid single spore cultures need to be crossed with a compatible homokaryon to produce mushrooms thus allowing phenotyping of mushroom traits. Plant breeders often start with an outcross sometimes using artificial or controlled pollination, followed by selfing to generate variation. Selfing in mushroom breeding is not often used since it has a number of drawbacks. The compatibility of mating between homokaryons is controlled by one or two, unlinked, loci (Kües 2015). Homokaryons are compatible if they differ in mating loci, a system directed to outbreeding. Selfing (crosses between homokaryotic SSI of one spore print) is restricted to 50 or 25% if compatibility is controlled by one or two unlinked loci, respectively. Mapping of traits linked to the mating type is impossible since this locus must remain heterozygous in order to produce mushrooms. Next to that, selfing also often leads to inbreeding depression in a number of fungal species including the button mushroom (Xu 1995). One or two backcrosses are usually possible without too much inbreeding symptoms but a third backcross might produce no fruiting bodies (A.S.M. Sonnenberg, unpublished observations). Backcrosses can thus be sed to some extent. Mushroom breeders use tester homokaryons compatible to the mating types present in a segregating haploid population of SSI. The advantage is that all hybrids thus generated share the genome of the tester line and segregation in phenotypes is due to genetic differences of the nucleus derived from the segregating SSI. The introduction of a new genome (the tester line) can, however, have an effect on the expression of traits resulting in some inconsistency in QTL mapping when assessing traits in the same population with different tester lines (Gao et al. 2016; Gao et al. 2015). The ability to recover the constituent nuclei of strains as homokaryons allows the estimation of a breeding value for traits of these nuclei comparable to what is done in some plant breeding programs (Griffing 1956; Shattuck et al. 1993). A selection of strains varying in the trait of interest can be protoplasted to recover the constituent nuclei of each heterokaryon. Mating of these homokaryons in all possible ways (a di-allel matrix) and testing these hybrids for the trait of interest allow the expression of the trait in different genetic backgrounds. That allows the assessment of the general combining ability (GCA) of a particular homokaryon/nucleus which is calculated as the deviation of the mean performance of a particular line from the overall mean of all crosses in the di-allel matrix (Gao et al. 2013). GCA is equivalent to the breeding value of an individual (Lynch and Walsh 1998) and the GCA of a line is a reflection of the number of loci with positive alleles for the trait and their effect (Adebambo 2011). Homokaryons with a positive effects can be used to stack favourable alleles of genes in a breeding program. Stacking multiple QTL in a cultivar can be facilitated by combining different subsets of QTL in each of the haplotypes of a receptor commercial cultivar using two backcrosses and selection with the appropriate markers. The resulting improved homokaryons can then be mated thus combining all QTL in one cultivar. The di-allel matrix can also identify parental lines with opposite GCA values which are good parents for the creation of a dedicated mapping population to study the genetics of traits of interest.

Selfing or cross breeding in plants leads to the formation of seeds that can be planted to study segregation of a trait. For mushroom breeding, the generation of hybrids is much more laborious. Each cross has to be generated separately by confronting both parental homokaryons on artificial media and selecting the heterokaryon after mating. The button mushroom has only one mating type (Mat-A) and the lack of a functional B-mating type results in the absence of nuclear migration after fusion of homokaryons and the absence of clamp connections, a structure that regulates the distribution of nuclei over the daughter cells (see Kothe 2001 for an overview of mating-type genes in mushroom strain improvement). The heterokaryon is thus only formed at the junction zone of the confronting homokaryons and microscopically indistinguishable from these homokaryons. Fortunately, heterokaryons usually grow faster than homokaryons facilitating selection of a potentially successful mating but each cross has in principle to be confirmed by using polymorphic genetic markers. For testing a phenotype, each cross has to be cultivated on substrate (compost). That requires the preparation of inoculum (spawn) for each cross and the cultivation of mushrooms under controlled conditions (Griensven Van 1988). Mushroom agronomic traits, such as yield, quality (scaling of cap, discoloration) but also extent of symptoms after infections are influenced by the quality of substrate and climate conditions. It is thus necessary to repeat trials. Studying a phenotypic variation in a segregating population is thus a laborious and costly task in which often a trade-off has to be made between the number of individuals to be tested and the number of replicates within a trial (Gao et al. 2016).

## Plant variety protection

**Fig. 3:**
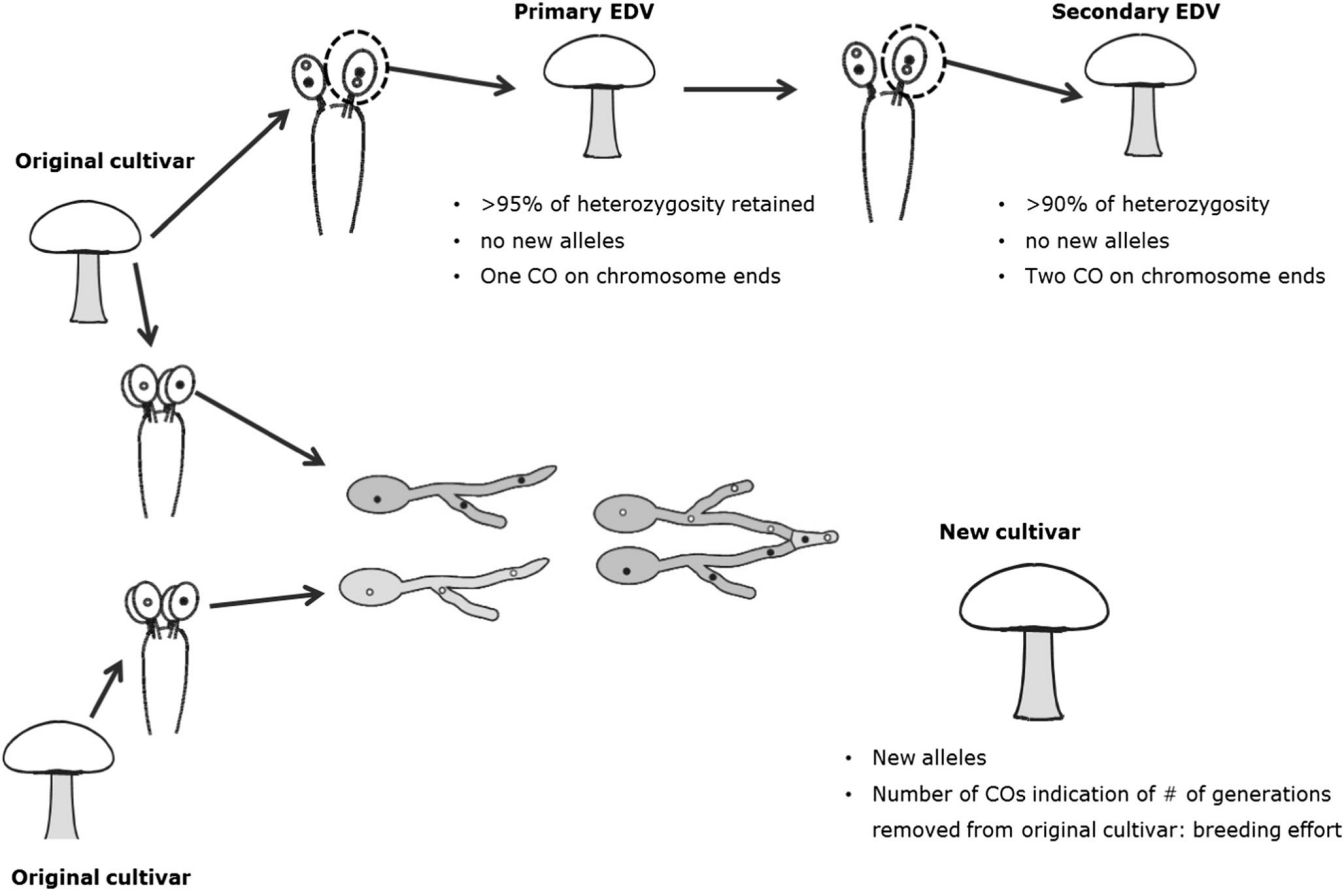
The whole genome sequencing can be used to precisely genotype a cultivar of button mushrooms and thus identify its genetic relationship to an original cultivar. The amount of retained heterozygosity and number of CO at chromosome ends can be used to see if a cultivar has been derived directly (primary EDV) or indirectly (secondary EDV) from the original variety. New alleles indicate that outbreeding has been used to generate a new cultivar based on the original variety and the number of CO relative to the original cultivar is an indication for the number of generations used (breeding effort)

Like plants, mushroom cultivars can be protected by plant variety right in most countries and several titles of protection have already been granted for mushroom strains (see for example http://www.cpvo.europa.eu/main/nl/). Breeding of a new cultivar in button mushrooms using outcrossing with the *bisporus* variety is a considerable job requiring much time and a large investment. Once generated, a very similar cultivar (an essentially derived variety (EDV)) can be made using fertile SSI derived from this first cultivar and can be marketed under different names. Such EDVs may outcompete the original cultivar and thus minimising the return of investment of the first breeder. This has been a practice for decades in the button mushroom industry as shown in previous paragraphs, and one of the reasons why only few truly different cultivars have been bred in the last 30 years. The generation of new cultivars via heterokaryotic SSI should thus be considered as EDVs as defined by UPOV (http://www.upov.int/) since this way of generation of new varieties meets all definitions of EDV: *mainly derived from the original variety*, *distinguishable from the original variety but*, *having otherwise strong resemblance to the original variety*. This applies only to EDVs generated from protected varieties. A definition of a new variety as an EDV requires the breeder to negotiate with the first breeder for a license to produce. The implementation and wide acceptation of this system will encourage investment in breeding of button mushrooms. For this purpose, a working group has been set up in 2012 to come to a consensus on a definition of EDV in general in edible mushroom breeding. The group consists of four breeding/spawn producing companies (Sylvan, Amycel, Limgroup and Lambert Spawn; representing 80% of the spawn market in Europe) and two research groups (INRA Bordeaux, France and Plant Breeding, Wageningen University and Research, the Netherlands). As a first target, the group has reached a consensus on considering fertile single spore cultures derived from a protected *A. bisporus* cultivar as an EDV (published on the different websites of all members of the working group). This consensus is supported by the main players in the mushroom industry in Europe and by the international scientific organisations (International Society for Mushroom Science and World Society for Mushroom Biology and Mushroom Products; Sonnenberg (2013)). The group has become recently part of the International Seed Federation (ISF) and will use the expertise of this organisation to generate a guideline for handling disputes on Essential Derivation in Mushroom Varieties. Relevant for EDV studies are the availability of global and EDV specific populations, accepted and widely available genetic marker technology and consensus on genetic similarity threshold (Bruins 2013). Proof for EDV might be more simple for mushrooms than for plants. The advantage of fungi over plants is their small genome and the ability to recover and genotype haplotypes. That allows the complete genotyping of haplotypes with the next generation sequencing technologies. Sonnenberg et al. (2016) have shown that whole genome sequencing can be used to correctly identify allele difference between varieties and exactly locate recombinations on each chromosome. That allows not only the assessment of genetic relationship between cultivars but can also reveal breeding history (Fig. 3). If a supposed new variety has lost some heterozygosity (<5%) and has otherwise identical alleles as the protected variety, this cultivar must have been derived from the protected variety via the isolation of heterokaryotic SSI. An EDV can be derived in this way directly from a variety or derived from a derivative. In the former case, usually one recombination is seen at chromosome ends relative to the original variety, while the latter will show on some chromosomes two nearby recombinations. Assuming that crossover interference is also active in the button mushroom, two nearby recombination spots can only be generated in separate (subsequent) generations. If new alleles are found relative to the protected variety, it has clearly been generated by outbreeding. The number of recombinations can be used as a count for the number of generations used to generate a new variety and thus estimate breeding effort. How to use these tools and where to put the threshold is now under debate.

## How to move on

The button mushroom *A. bisporus* is one of the most cultivated mushroom species with an annual crop value exceeding $3.2 billion worldwide in 2009 (Sonnenberg et al. 2011) and probably now more than $4 billion considering the large expansion in China. The progress made in the last decades in yield and quality is mainly due to optimising substrate and climate conditions and not by breeding improved cultivars. New cultivars can have a large impact for a crop in the food market as has been shown by breeders of tomato and sweet pepper varieties. A similar effect could be generated for button mushroom with development of new cultivars for the primary producers but especially directed to consumer benefits (taste, nutritional quality, shelf life, etc.). Despite the progress made in the last 20 years, mushroom breeding is still lagging behind plant breeding. Especially for breeding button mushrooms, technical hurdles have to be tackled in order to make it more efficient. The introduction of the tetrasporic trait and more even recombination along chromosomes of the var. *burnettii* into breeding stock of the var. *bisporus* would facilitate breeding enormously. Next to that, plant breeders derive their breeding materials from advanced (elite) germplasm, improved germplasm, landraces or wild lines (Acquaah 2015). Because of lack of advanced breeding stock, (button) mushroom breeders are forced to use wild germplasm for improvement or introduction of new traits. Such breeding programs often start with hybrids low in most of the agronomic traits and need a substantial backcrossing to restore the desired agronomic traits. This takes time and so far has not led to commercially viable cultivars. A joint effort similar to what has been done in the past for plant breeders can generate a platform of advanced breeding stock which can be used by each breeder to generate its own new cultivars. Such pre-breeding programs should include thorough and standardised phenotyping of important traits in collections and a subsequent transfer of these traits into an advanced breeding stock which has an acceptable yield and quality and can be used to develop new cultivars within foreseeable future. As important as solving these technical hurdles is the need to improve protection of new varieties. Without a proper protection, no substantial investment will be done in breeding and consequently not in breeding research. The general acceptance that selection of heterokaryotic SSI of *A. bisporus* should be considered as generating EDVs will certainly help. A wider definition of EDV for button mushrooms is needed and for edible mushrooms in general. Last but not least, although a protection of new mushroom cultivars is possible by plant variety rights, it still seems the common practice to copy protected cultivars and market them under different names (A.S.M. Sonnenberg, personal observations) facilitated by the ease of generating tissue cultures from marketed mushrooms or generating spawn from commercial spawn lots. Other than Western countries, breeding of mushrooms in China and Korea is mainly financed by governments. In China, two new button mushrooms have been generated and these dominate now the Chinese market (Meiyan et al. 2009; Wang et al. 1995). Protection of new varieties is also an issue in China, but since private investment in breeding is almost absent, infringement is a lesser issue than in Western countries. Although the present-day sequencing tools can be used to irrefutably prove that a strain has been copied, it seems not easy to generate proof of infringement due to limitations in freedom to sample infringers and lack of track-and-trace of produce. Legal bodies are now working on this issue. Important is also to increase the awareness in the mushroom industry that infringement of protected cultivars hampers the development of new varieties. Despite the technical and legal hurdles to take, this review shows that considerable progress has been made in our knowledge on breeding the button mushroom. A number of prerequisites have been met to improve traits in button mushroom varieties. Introduction of these traits in existing cultivars will benefit the primary producers in generating food products in a more competitive and sustainable way. It offers also opportunities for innovation in the final product, i.e. the mushrooms, in order to enlarge the position of edible mushroom in food markets.

## Electronic supplementary material

ESM 1 (PDF 348 kb).
