# Supplementary material for: Developments in breeding of Agaricus bisporus var. bisporus: progress made and technical and legal hurdles to take
Source: Appl Microbiol Biotechnol. 2017 Jan 28;101(5):1819–29. doi: 10.1007/s00253-017-8102-2 (PMC5309338; doi:10.1007/s00253-017-8102-2)
Supplement: Supplementary file 1 — (PDF 348 kb). [file 253_2017_8102_MOESM1_ESM.pdf]

Applied Microbiology and Biotechnology

Developments in breeding of *Agaricus bisporus* var. *bisporus*: Progress made and technical and legal hurdles to take.

Anton S.M. Sonnenberg[1], Johan J. P. Baars[1], Wei Gao[2], Richard G.F. Visser[1]

[1] Plant Breeding, Wageningen University & Research , 6708PB, Wageningen, the Netherlands

[2] Institute of Agricultural Resources and Regional Planning of CAAS, Zhongguancun South Street 12, 100081, Beijing, China

Corresponding author: anton.sonnenberg@wur.nl; +31317-481336

Supplementary data: Genotyping strains using SNPs

For the analysis of the genetic relationship between strains of the collection, 117 SNP were used with the KASPar technology. First, five strains were selected know not to be genetically closely related (Table S1). Of these strains, one constituent nucleus was isolated by protoplasting and sequenced (Illumina 2000). SNPs were generated relative to the reference strain H97 (constituent nucleus of the commercial cultivar Horst U1). For each chromosome, 1 to 3 SNPs were selected unique for one of the homokaryons (Table S2; see sheet S1 for the sequences of these SNPs). The similarity among the individual heterokaryons was scored by the presence or absence of each SNP, where the presence is represented by 1 or 2 SNP (the latter means presence in both nuclei). The data were transformed to nucleotide data (A & T) and a similarity matrix was generated using Mega 5.1 (Tamura et al., 2007). The Maximum Likelihood statistical method of Mega 5.1 was subsequently used to generate a phenogram (Statistical method: Maximum Likelihood; Bootstrap 500). Graphs were generated using Archeopteryx version 0.972 (Han & Zmasek, 2009). The sequence of the target SNPs and the strains used for genotyping are listed in the supplementary tables (Tables S3 and S4).

Table S1

	Constituent nucleus of:	Strain type	Life cycle
H39	Horst U1	Present-day white hybrid	*bisporus*
bisp 141_3	bisp 141	Brown wild isolate	*burnettii*
MES 09143	bisp 53	Brown wild isolate	*bisporus*
Z8	bisp 170	White wild isolate	*bisporus*
JB137-S8	JB137	Brown wild isolate	*burnettii*

Table S2.

Strains	Ch_1	Ch_2	Ch_3	Ch_4	Ch_5	Ch_6	Ch_7	Ch_8	Ch_9	Ch_10	Ch_11	Ch_12	Ch_13	Totals
bisp141_3	2	2	2	2	2	2	1	1	2	3	2	2	2	25
H39	2	2	2	2	2	2	2	2	2	2	2	2	2	26
JB137	2	2	2	2	2	2	2	2	2	3	3	3		27
MES09143	2	2	2	2	2	2	1	2	2	3	2	2	1	25
Z8	1	2		1	1		2	2	1	1	1	1	1	14
Totals	9	10	8	9	9	8	8	9	9	12	10	10	6	117

Legend to Supplementary tables.

Table S1. Homokaryons (isolated as a constituent nucleus from heterokaryons by protoplasting) used to generate SNPs for genotyping the collection of *Agaricus bisporus* strains in the Plant Breeding collection.

Table S2. Number of SNPs selected for each chromosome and unique for one of the strains used (Column 1).

Table S3. Sequences of the SNPs used to genotype the collection of A. bisporus strains.

Table S4. Lists of A. bisporus strains genotypes by SNPs markers described in Table S3.
